# Prognostic role of IDH mutations in gliomas: a meta-analysis of 55 observational studies

**DOI:** 10.18632/oncotarget.4008

**Published:** 2015-07-16

**Authors:** Liang Xia, Bin Wu, Zhiquan Fu, Fang Feng, Enqi Qiao, Qinglin Li, Caixing Sun, Minghua Ge

**Affiliations:** ^1^ Zhejiang Cancer Hospital, Hangzhou, Zhejiang Province, China; ^2^ Shanghai hospital of integrated traditional and western medicine, Hongkou District, Shanghai, China

**Keywords:** IDH mutations, prognosis, glioma

## Abstract

**Background:**

IDH (Isocitrate dehydrogenase) mutations occur frequently in gliomas, but their prognostic impact has not been fully assessed. We performed a meta-analysis of the association between IDH mutations and survival in gliomas.

**Methods:**

Pubmed and EMBASE databases were searched for studies reporting IDH mutations (IHD1/2 and IDH1) and survival in gliomas. The primary outcome was overall survival (OS); the secondary outcome was progression-free survival (PFS). Hazard ratios (HR) with 95% confidence interval (CI) were determined using the Mantel-Haenszel random-effect modeling. Funnel plot and Egger's test were conducted to examine the risk of publication bias.

**Results:**

Fifty-five studies (9487 patients) were included in the analysis. Fifty-four and twenty-seven studies investigated the association between IDH1/2 mutations and OS/PFS respectively in patients with glioma. The results showed that patients possessing an IDH1/2 mutation had significant advantages in OS (HR = 0.39, 95%CI: 0.34–0.45; *P* < 0.001) and PFS (HR = 0.42, 95% CI: 0.35–0.51; *P* < 0.001). Subgroup analysis showed a consistent result with pooled analysis, and patients with glioma of WHO grade III or II-III had better outcomes.

**Conclusions:**

These findings provide further indication that patients with glioma harboring IDH mutations have improved OS and PFS, especially for patients with WHO grade III and grade II-III.

## INTRODUCTION

Gliomas account for more than 50% of all central nervous system (CNS) tumors [1]. Glioblastomas remain among the deadliest human tumors in spite of recent advances in both diagnostic modalities and therapeutic strategies. Indeed, the 5-year survival rate in patients with glioblastoma is among the lowest for all neoplasms. Indeed, in patients with glioblastoma multiforme, the median survival is 9–12 months [2]. All gliomas are classified from grade I to grade IV according to the 2007 WHO Classification of Cancers of the CNS [3]. Established clinicopathological prognostic factors such as WHO grade, age at diagnosis, performance status, cognitive function, histologic characteristics, and extent of surgical resection had been shown to inadequately predict the clinical outcomes of gliomas [4]. A number of molecular markers with important roles in the formation and progression of gliomas have been identified recently. However, only a few have practical value in the clinical setting [5]. Therefore, identification of novel prognostic markers has a substantial clinical impact on the future management of gliomas.

Isocitrate dehydrogenase 1/2 (IDH1/2) catalyze the oxidative carboxylation of isocitrate to α-ketoglutarate, resulting in the reduction of nicotinamide adenine dinucleotide phosphate (NADP) to NADPH, which is necessary for the regeneration of reduced glutathione, which is the main antioxidant in cells [6]. Parsons (2008) firstly proposed the presence of mutations in the active site of IDH1/2 in most low-grade gliomas and secondary high-grade gliomas [7]. Several subsequent researches further supported his conclusions and have found that IDH1/2 might be a prognostic factor since patients with a glioma harboring an IDH mutation show significantly better survival than those with a wild-type IDH glioma [8–12]. The latest study with a large sample (1305 patients) and the preceding meta-analysis including 12 studies (2,190 patients) published in 2013 also supports this association [13, 14]. However, potential heterogeneity was indicated among these studies, which did not explore the prognostic value of IDH mutations based on different clinical and pathological features.

Therefore, the aim of the present study was to synthesize comprehensively the available evidence on the effects of IDH mutations on survival in patients with gliomas.

## MATERIALS AND METHODS

### Search strategy and study selection

A comprehensive search of the English medical literature was conducted on studies evaluating the effect of IDH1/2 mutation on the survival of patients with glioma. Pubmed and EMBASE were searched by using the terms (“Glioma” or “Glioblastoma”) and (“Isocitrate Dehydrogenase” or “Isocitrate Dehydrogenase-1” or “IDH1” or “IDH2” or “IDH”) and (“Mutation”) and (“Survival” or “Mortality” or “Prognosis”). The literature search was executed in November 2014. Detailed search strategies for both databases are shown in Supplementary Appendix 1. Furthermore, we manually searched references in pertinent studies that were identified during the screening processes.

All candidate studies were reviewed by two independent reviewers (Xia L and Li QL). Discrepancies were resolved by discussion. Our search was initially narrowed based on the title followed by the abstract, and finally full papers were reviewed if they were categorized as relevant studies. All of the references from review papers and original reports were examined for further relevant studies. Including criteria for selecting the studies for our analysis were: (i) the diagnosis of glioma was made by pathological examination; (ii) correlation of mutant IDH1/2 with OS or PFS was reported; (iii) the study was the most recent or comprehensive report if the same group or author reported results procured from the same glioma patient population in more than one article; (iv) the papers that were not directly providing hazard ratios (HRs)/odds ratios (ORs) and 95% confidence intervals (CI) were kept if we could rebuild them using the *P* values and other data reported; (v) studies had more than 40 patients; and (vi) studies published in English.

### Data extraction

Two reviewers (Xia L and Li QL) independently extracted data from included studies: first author's name, year of publication, country of the population studied, number of patients, patient age, WHO grade, percentage of IDH mutant, IDH mutation type, primary or secondary, mutation detection method, treatment regimen, survival data including OS and PFS, follow-up period, survival analysis, and adjusted variables. OS (overall survival) was defined from the medical treatment until death or last follow-up. PFS (progression free survival) was calculated as the interval between the date of treatment and the detection of the recurrence or death from any cause. Disagreements were addressed by discussion with a third reviewer (Li Qinglin) until the two first reviewers reached a consensus or by contacting experts if necessary.

### Quality assessment of primary studies

Quality assessment of included primary studies was independently performed by two reviewers (Xia L and Li QL) using the Newcastle-Ottawa Quality Assessment Scale (NOS) [15]. NOS scores of  ≥ 6 were defined as high-quality studies. Any disagreement was solved by discussion.

### Statistical analysis

The Stata 12.0 statistical software (Stata Corporation, College Station, TX, USA) was used to perform the meta-analysis. Hazard ratio (HR) and 95%CI were obtained directly from each article or from an estimation of the Kaplan-Meier survival curves using the methods by Parmar et al [16]. An HR < 1 indicated a better prognosis in patients with glioma and IDH mutation, whereas an HR > 1 implied a poor prognosis. If several HR estimates were presented in the same study, we chose the most powerful one (multivariate analysis was superior to univariate analysis, and univariate analysis was superior to unadjusted Kaplan-Meier analysis).

The Cochrane's Q statistic was used to evaluate the heterogeneity of the primary studies. I2 > 50% is defined as a measure of severe heterogeneity. The random-effects model, which is generally more conservative, was chosen. In addition, subgroup analyses were performed to investigate the potential causes of heterogeneity according to IDH1/2 and IDH1 mutations, study origin, sample size, follow-up period, patient age, mutation detection method, survival analysis and WHO grade.

Publication bias was first assessed by visual judgment of a funnel plot, and then performed for each of the pooled study groups using the Egger's test. All *P* values were two-sided and the significance level was set at 0.05.

## RESULTS

### Study selection procedure

**Figure 1:**
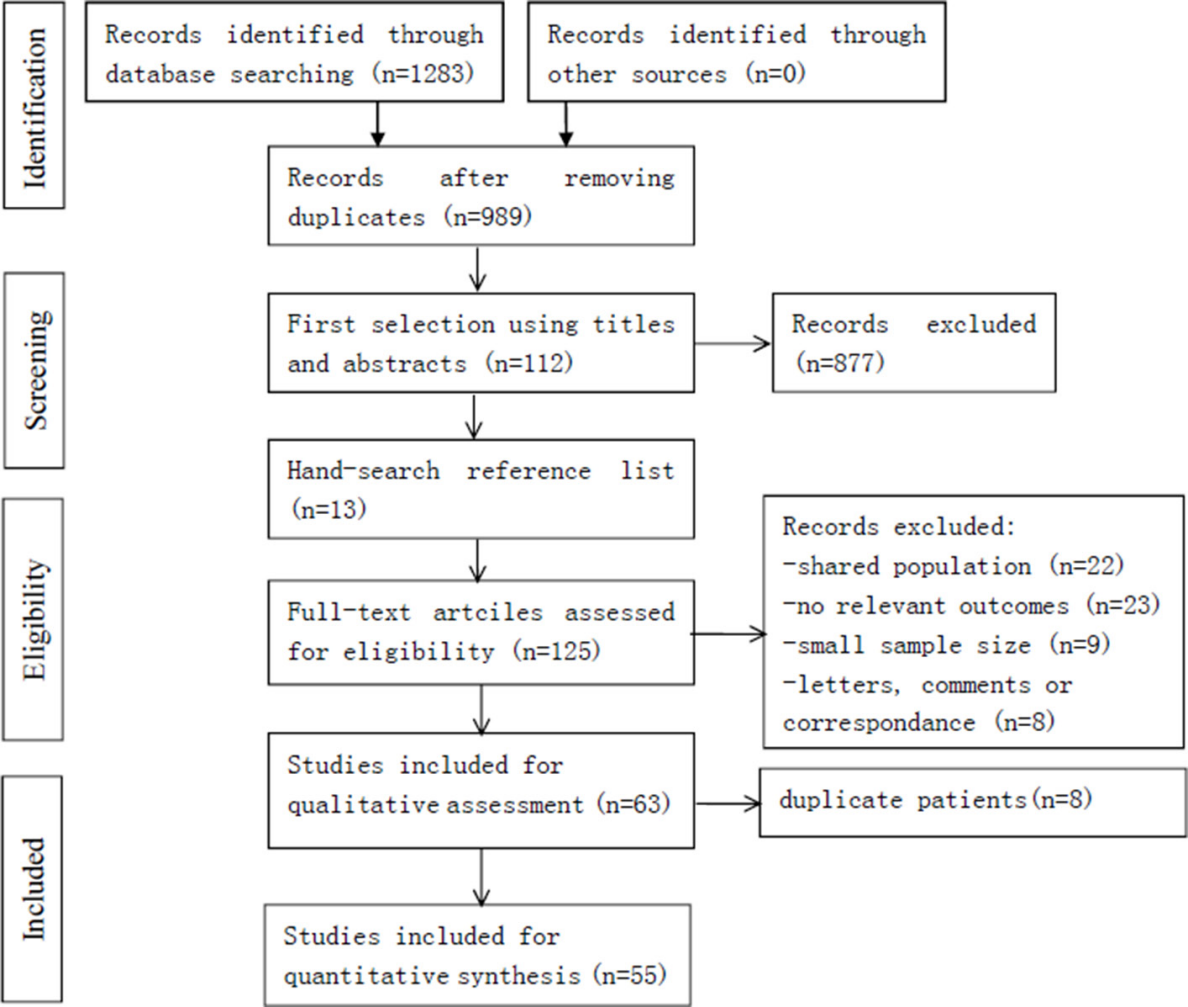
Flowchart of the included studies

The study selection procedure is presented in Figure 1. In the initial literature search, 1283 studies were relevant to the search terms. Of which, we excluded 294 studies because of overlapping data sets. Then, 877 studies were ruled out because of apparent irrelevance when reading the title and/or abstract. An additional 13 relevant studies were included from reference lists. By reading through the full texts of the remaining studies, 62 studies were excluded (22 studies shared an identical population, 23 studies had no relevant outcomes, nine studies were with small sample size, and eight articles were letters, comments or correspondence). Moreover, 8 studies were ruled out due to the duplicate patients. Finally, 55 articles were left with sufficient data for extraction.

### Study characteristics

Characteristics of included studies are summarized in Supplementary Table 1. Fifty-five studies were included: 9 studies from America, 17 from Asia and 29 from Europe [8–11, 13, 17–66]. A total of 9487 patients were included, and the range of mean age was 36 to 64.3 years, and the sample size was 40 to 1305 patients. Twelve studies evaluated grade II gliomas, six examined grade III gliomas, 12 examined grade IV glioma, four evaluated grades I-IV tumors, three evaluated grades II-III tumors, five examined grades III-IV gliomas and 13 evaluated grades II-IV gliomas.

### IDH 1 and IDH1/2 mutations and OS in gliomas

**Figure 2:**
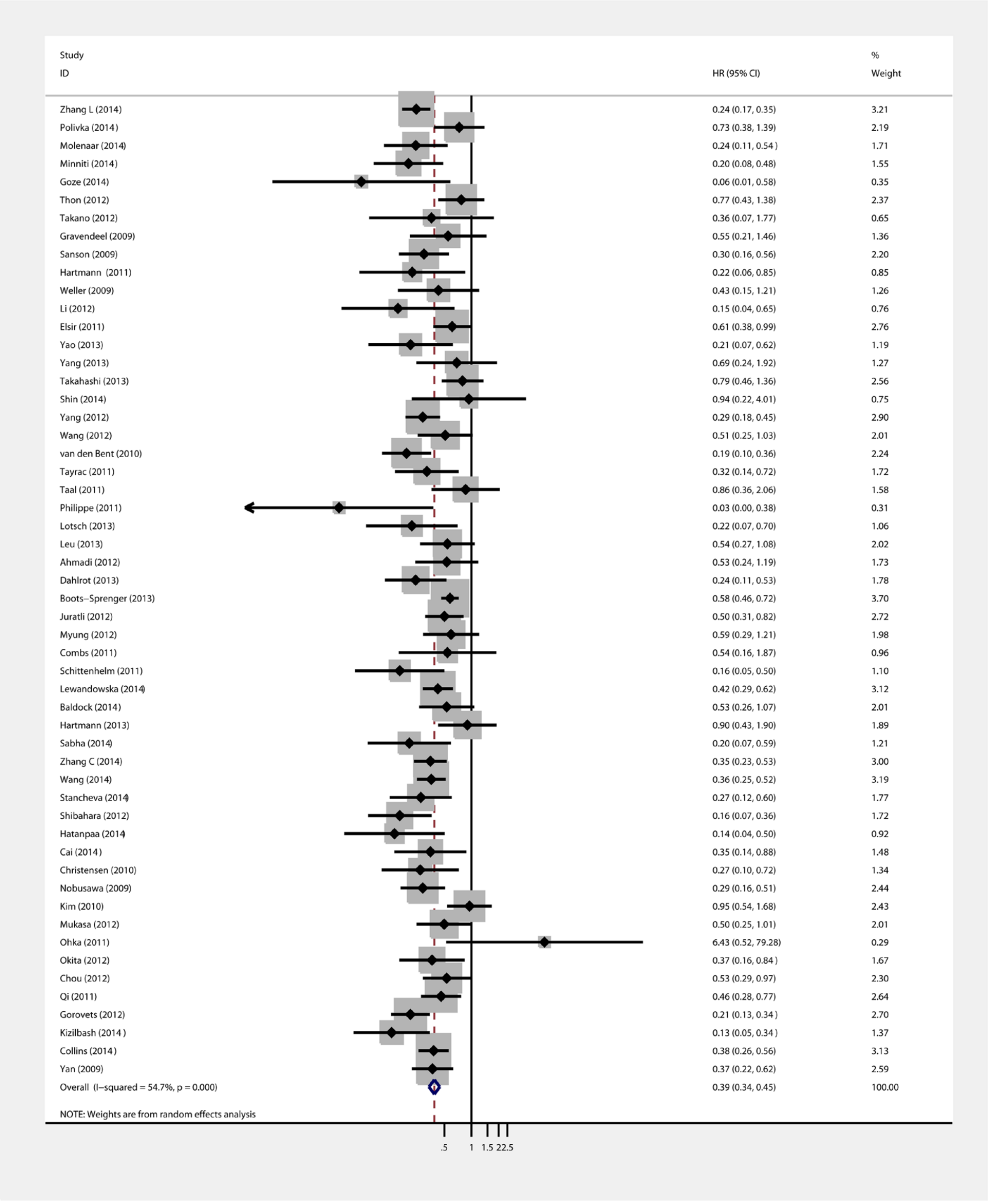
Forest plot of HR and 95%CI of the association between IDH1/2 mutations and OS of gliomas

Fifty-four of 55 studies investigated the association between IDH1/2 mutations and OS of patients with glioma. The combined analysis showed that upon comparing patients without IDH1/2 mutations, patients possessing a mutation had a significant OS advantage (HR = 0.39, 95%CI: 0.34–0.45; *P* < 0.001) (Table 1, Figure 2). Since statistically significant heterogeneity was observed between the studies (I2 = 54.8, *P* < 0.001), further subgroup analyses were conducted according to the IDH1/2 and IDH1 mutations, study origin, sample size, follow up period, patient age, mutation detection method, survival analysis and WHO grade. Regarding the publication bias in the studies, we found no funnel plot asymmetry. Furthermore, the results of the Egger's test did not show any evidence of publication bias (*P* = 0.230 for OS, Figure 4A).

**Table 1:** Subgroup analyses of the relationships between IDH mutations and overall survival or progression-free-survival

Comparison variables	Overall survival			Progression-free survival		
	Number of studies, Heterogeneity		Interaction	Number of studies, Heterogeneity		Interaction
	(I^2^ statistics; %)	HR 95%CI, *P* value	*P* value	(I^2^ statistics; %)	HR 95%CI, *P* value	*P* value
Total	54 (54.7)	0.39 (0.34 to 0.45), < 0.001	NA	27 (59.15)	0.42 (0.35 to 0.51), < 0.001	NA
***Mutation***			0.17			0.55
IDH1	34 (58.7)	0.42 (0.35 to 0.50), < 0.001		16 (71.8)	0.50 (0.36 to 0.68), < 0.001	
IDH1/2	20 (58.3)	0.37 (0.30 to 0.47), < 0.001		11 (78.4)	0.42 (0.33 to 0.53), < 0.001	
***Origin country***						
North America	9 (45.2)	0.30 (0.22 to 0.43), < 0.001	0.28	1 (NA)	0.15 (0.05 to 0.42), < 0.001	0.03
Asian	16 (52.6)	0.40 (0.32 to 0.50), < 0.001		12 (74.2)	0.44 (0.32 to 0.60), < 0.001	
Europe	29 (56.3)	0.42 (0.36 to 0.48), < 0.001		14 (62.1)	0.54 (0.45 to 0.64), < 0.001	
***Sample size***						
≥200	16 (52.9)	0.41 (0.36 to 0.48), < 0.001	0.34	8 (67.6)	0.46 (0.38 to 0.55), < 0.001	075
<200	38 (54.8)	0.37 (0.31 to 0.45), < 0.001		19 (77.2)	0.49 (0.34 to 0.69), < 0.001	
***Follow up period***						
Referred	20 (58.3)	0.37 (0.30 to 0.47), < 0.001	0.64	13 (81.3)	0.45 (0.30 to 0.66), < 0.001	0.77
No Referred	34 (51.0)	0.40 (0.35 to 0.46), < 0.001		14 (60)	0.50 (0.42 to 0.59), < 0.001	
***Median/mean age y***						
> 45	18 (52.2)	0.38 (0.31 to 0.46), < 0.001	0.15	8 (78.2)	0.41 (0.30 to 0.55), < 0.001	0.10
< 45	19 (56.7)	0.46 (0.38 to.0.57), < 0.001		13 (77.8)	0.58 (0.43 to 0.80), 0.001	
NR	17 (43.4)	0.34 (0.28 to 0.42), < 0.001		8 (30.0)	0.38 (0.30 to 0.49), < 0.001	
***Mutation detection***						
Direct sequencing	31 (52.9)	0.42 (0.36 to 0.48), < 0.001	0.07	15 (77.4)	0.50 (0.41 to 0.62), < 0.001	0.35
Pyro-sequencing	9 (52.9)	0.42 (0.29 to 0.59), < 0.001		5 (63.3)	0.49 (0.30 to 0.80), 0.005	
Immunohistochemistry	12 (53.9)	0.35 (0.25 to 0.49), < 0.001		6 (66.3)	0.33 (0.18 to 0.60), < 0.001	
NR	2 (21.1)	0.23 (0.15 to 0.36), < 0.001		1 (0.0)	0.36 (0.24 to 0.54), < 0.001	
***Survival analysis***						
Multivariate	43 (52.6)	0.37 (0.32 to 0.42), < 0.001	0.04	24 (70.9)	0.44 (0.37 to 0.52), < 0.001	0.10
Others	11 (42.6)	0.48 (0.39 to 0.58), < 0.001		3 (86.6)	1.07 (0.37 to 3.07), 0.902	
***WHO grade***						
II	17 (59.5)	0.45 (0.35 to 0.59), < 0.001	0.0003	13 (60.4)	0.66 (0.48 to 0.90), 0.009	0.006
III	11 (10.1)	0.27 (0.22 to 0.32), < 0.001		7 (85.1)	0.22 (0.12 to 0.42), < 0.001	
IV	20 (46.1)	0.49 (0.40 to 0.60), < 0.001		9 (75.2)	0.65 (0.41 to 1.05 ),0.078	
II-III	4 (62.4)	0.28 (0.14 to 0.54), < 0.001		3 (25.0)	0.32 (0.21 to 0.48), < 0.001	
III-IV	5 (58.6)	0.40 (0.28 to 0.57), < 0.001		2 (36.1)	0.40 (0.25 to 0.66), < 0.001	
II-IV	8 (0.0)	0.39 (0.32 to 0.49), < 0.001		5 (8.0)	0.41 (0.34 to 0.49), < 0.001	

### IDH1/2 mutations and PFS in gliomas

**Figure 3:**
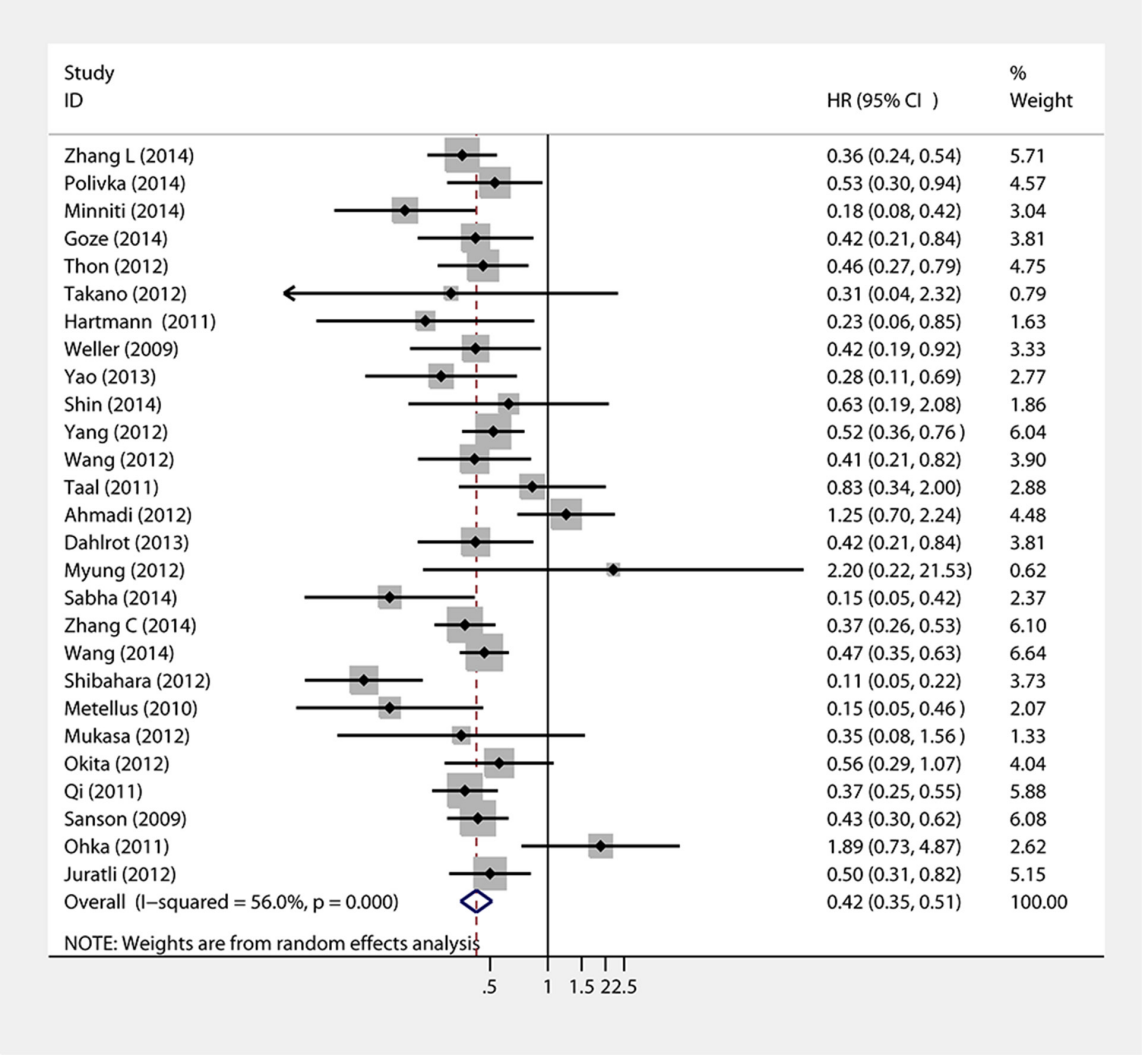
Forest plot of HR and 95%CI of the association between IDH1/2 mutations and PFS of gliomas

**Figure 4:**
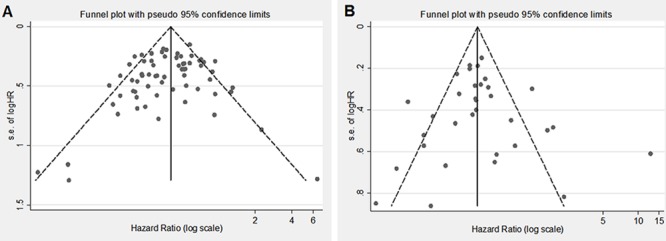
Egger's funnel plot for the publication bias test of the IDH1/2 mutations and OS A. or PFS B. of human gliomas

Twenty-seven studies provided data concerning the association between IDH1/2 mutations and PFS of glioma patients. The combined analysis of the included studies showed that upon comparing patients without IDH1/2 mutations, patients possessing a mutations status had a significant PFS advantage (HR = 0.42, 95% CI: 0.35–0.51; *P* < 0.001) (Table 1, Figure 3). Statistically significant heterogeneity was observed between the studies (I2 = 59.2). No funnel plot asymmetry was found, and the Egger's test did not show any evidence of publication bias (*P* = 0.783 for PFS; Figure 4B).

### Subgroup analysis

Table 1 presents the subgroup analyses of the relationship between IDH mutations and OS and PFS. There was no difference in OS and PFS between patients with IDH1 and IDH1/2 mutations (*P* = 0.17 and *P* = 0.55, respectively). Therefore, these two groups of patients were analyzed together in the next subgroup analyses. Country of origin had no impact on OS (*P* = 0.28), but was associated with a better PFS in North America (HR = 0.15, 95%CI: 0.05–0.42) compared with Asia (HR = 0.44, 95%CI: 0.32–0.60) and Europe (HR = 0.54, 95%CI: 0.45–0.64) (*P* = 0.03). Sample size of the study, follow-up period, age, and mutation detection method had no effect on OS and PFS. Multivariate survival analysis led to a better OS (*P* = 0.04) compared with other methods, but had no effect on PFS. Finally, WHO grade had an impact on both OS (*P* = 0.0003) and PFS (*P* = 0.006). Supplementary Table S2 presents the OS and PFS for each individual study.

## DISCUSSION

### Principal findings

The present meta-analysis showed that patients with gliomaharboring IDH1/2 (including IDH1 mutation) had a significant OS and PFS advantage over those without IDH1/2 mutations. Specifically, harboring a mutation in IDH1/2 mutation reduced overall mortality by 61% and progression-free mortality by 58% compared with the wild-type genes. At the same time, the summary HRs across studies calculated for each subgroup did not alter substantially the OS and PFS results for IDH1/2, despiting subgroup “Others” of “survival analysis” in OS and “IV” of “WHO grades” in PFS. Finally, results suggest that the methodology used to assess IDH mutation is not very important. Indeed, sequencing is more sensitive than immunochemistry but given that more than 90% of IDH mutations are IDH1 mutations and that in these mutations the R132H one is found in more than 90% of cases, immunochemistry is a reliable diagnosis technique due to its high sensitivity and specificity [32, 67].

### Potential tumorigenesis mechanism and prognostic value of IDH in glioma

IDH1 and IDH2 genes have become a focus for research aimed at understanding the biology of gliomas [6]. Mutations in these genetic loci, first discovered in 2008, occur in a large proportion of low-grade gliomas and secondary glioblastomas [7]. Researchers have found that IDH mutationsare relatively glioma-specific and are likely to be a direct cancer driver in the early stage of gliomagenesis [6]. Firstly, IDH mutations produce 2-hydroxyglutarate (2-HG) and it functions as a possible oncometabolitecontributing to the tumorigenesis and progression of gliomas [68]. Studies have shown that 2-HG is equivalent to α-ketoglutarate in structure, and competitively suppresses the activity of dioxygenases [69]. Thus, suppression of dioxygenases by 2-HG is believed to be one of the mechanisms through which IDH mutations lead to the pathogenesis of gliomas. Secondly, IDH1 mutant can inhibit the activity of the prolyl hydroxylase and hence the stability of the hypoxia-inducible factor 1α (HIF1α) by reduced production of α-ketoglutarate and increased production of 2-HG [70]. Finally, HIF1αcan activate a series of target genes that might promote glioma cell growth, invasion, angiogenesis, and metastasis [71]. Thirdly, IDH1 mutation may be closely linked to the epigenetic program [72]. The Cpg Island Methylator Phenotype (CIMP) is a powerful determinant of glioma pathogenicity. Turcan et al [72] study has shown that IDH1 mutation contributes to the establishment of glioma-CIMP by rebuilding the methylome. Their findings indicated that IDH mutations might be a molecular basis of CIMP in gliomas, providing a direction for understanding oncogenesis in human glioma.

IDH mutations may serve as prognostic factors and are strongly correlated with good prognosis in patients with glioma. Many studies have shown that the median OS of patients whose glioblastoma harbor an IDH1 and IDH2 mutations was significantly longer than that of patients whose glioblastoma harbor wild-type IDH [30, 54, 73]. In addition, IDH mutations are associated with better prognosis in patients with anaplastic astrocytoma [57]. Subsequent multivariate analysis confirmed that IDH1 mutation may be an independent favorable prognostic marker in glioblastoma and anaplastic glioma after adjustment for other genomic profiles and treatment modalities [74]. The present meta-analysis further confirms the prognostic value of IDH mutations based on different clinical and pathological featuresreported in 55 observational studies (9487 patients).

### Comparison with other studies

The results from this meta-analysis are broadly consistent with two previously published studies, a meta-analysis of nine studies in glioblastoma [75] and a meta-analysis of twelve studies [14] in adults with different grades of gliomas. These two studies found that IDH1/2 mutations significantly improved the outcomes for patients with glioma. However, in contrast to the two previous meta-analyses, the present meta-analysis included 55 independent publications, with 9487 glioma cases, which should meaningfully increase the statistical power and accurately estimate the effect of IDH1/2 mutations on the prognosis of patients with glioma. In addition, we also conducted more detailed subgroup analyses based on more different clinical and pathological features to systematically evaluate the prognostic effect of IDH mutation and identify potential sources of heterogeneity. Especially, subgroup analyses according to WHO grade revealed that the presence of IDH mutations was associated with a better outcome in patients with WHO grade III and grade II-III. Furthermore, in the present meta-analysis, most of the HRs from the included studies were from multivariate analyses adjusting for confounding factors. Indeed, the adjusted HRs are more accurate than the unadjusted HRs since they reduce the risk of bias from other possible confounding factors. Hence, the findings from the present meta-analysis provide strong evidence that IDH mutations carry a very strong prognostic significance for PFS and OS in patients with glioma. Finally, a significant insight from this research is that our included study population was mainly from three continents including eighteen countries. Therefore, the present study ensures an extensive value for the use of IDH mutations in the prognosis of patients with glioma.

### Limitations

The present meta-analysis may have several limitations that need to be addressed. First, in spite of the comprehensive search strategy, we cannot avoid the possibility of having missed relevant studies, in particular studies published in languages other than English. There may have been negative studies that were never published as full-length articles, and the original data of several studies could not be obtained. Secondly, we based our analyses on retrospective studies rather than prospective studies, so that it is hard to effectively avoid recall and selection biases. Thirdly, several of the HRs in the included studies were from rough estimates of Kaplan-Meier survival curves, so the results may be not so accurate. Fourthly, most of the HRs were from multivariate analyses by adjusting for confounding factors; however, there were different confounding factors in different included studies. Therefore, the merged HRs have a degree of heterogeneity. Fourthly, the proportion of histological subtypes varies between studies, suggesting a potential diagnostic bias between centers. Finally, evident heterogeneity existed for several outcomes that could not be explained substantially by our present subgroups. This limits our understanding of the association in various settings and restricts the general is ability of our findings.

## CONCLUSION

In conclusion, this meta-analysis provides more strong evidence that IDH1/2 mutations is an independent prognostic factor for patients with different grades of glioma and that IDH1/2 mutations is associated with improved OS and PFS, especially for patients with WHO grade III and grade II-III.

## SUPPLEMENTARY FIGURE AND TABLE




