# Supplementary material for: Prognostic role of IDH mutations in gliomas: a meta-analysis of 55 observational studies
Source: Oncotarget. 2015 Jul 16;6(19):17354–65. doi: 10.18632/oncotarget.4008 (PMC4627313; doi:10.18632/oncotarget.4008)
Supplement: Supplementary file 1 [file oncotarget-06-17354-s001.pdf]

SUPPLEMENTARY FIGURE AND TABLE

PUBMED SEARCH (20 Sep 2014)

Search (((((“Glioblastoma”[Mesh]) OR “Glioma”[Mesh]) OR ((((glioma*[Title/Abstract]) OR glioblastoma*[Title/Abstract]) OR glioblastoma multiforme[Title/Abstract]) OR Astrocytoma*[Title/Abstract]))) AND ((((“Isocitrate Dehydrogenase”[Mesh]) AND “Mutation”[Mesh])) OR (((((((((((isocitrate dehydrogenase[Title/Abstract]) OR Isocitrate Dehydrogenase-1[Title/Abstract]) OR Isocitrate Dehydrogenase 1[Title/Abstract]) OR IDH[Title/Abstract]) OR IDH1[Title/Abstract]) OR IDH-1[Title/Abstract]) OR IDH2[Title/Abstract]) OR IDH-2[Title/Abstract]) OR Isocitrate Dehydrogenase-2[Title/Abstract]) OR Isocitrate Dehydrogenase 2[Title/Abstract])) AND mutation*[Title/Abstract]))) AND ((((“Survival”[Mesh]) OR “Mortality”[Mesh]) OR “Prognosis”[Mesh]) OR ((((((Prognos*[Title/Abstract]) OR outcome*[Title/Abstract]) OR survival[Title/Abstract]) OR mortality[Title/Abstract]) OR recurren*[Title/Abstract]) OR predict*[Title/Abstract]))

Results: 466

Embase Session Results (20 Sep 2014)

No.	Query	Results
#17	#4 AND #11 AND #16	817
#16	#12 OR #13 OR #14 OR #15	3578470
#15	prognos*:ab,ti OR outcome*:ab,ti OR survival:ab,ti OR mortality:ab,ti OR recurren*:ab,ti OR predict*:ab,ti AND [embase]/lim	3091168
#14	‘prognosis’/exp	458131
#13	‘mortality’/exp	671502
#12	‘survival’/exp	626181
#11	#7 OR #10	2295
#10	#8 AND #9	1981
#9	mutation:ab,ti OR mutations:ab,ti AND [embase]/lim	459320
#8	idh:ab,ti OR idh1:ab,ti OR ‘idh 1’:ab,ti OR ‘isocitrate dehydrogenase’:ab,ti OR ‘isocitrate dehydrogenase-1’:ab,ti OR ‘isocitrate dehydrogenase 1’:ab,ti OR ‘isocitrate dehydrogenase-2’:ab,ti OR ‘isocitrate dehydrogenase 2’:ab,ti OR idh2:ab,ti OR ‘idh 2’:ab,ti AND [embase]/lim	4615
#7	#5 AND #6	772
#6	‘mutation’/exp	762631
#5	‘isocitrate dehydrogenase’/exp	4851
#4	#1 OR #2 OR #3	99504
#3	glioma*:ab,ti OR glioblastoma*:ab,ti OR ‘glioblastoma multiforme’:ab,ti OR astrocytoma:ab,ti AND [embase]/lim	64273
#2	‘glioblastoma’/exp	37487
#1	‘glioma’/exp	88120

Supplementary Table S1: Main characteristics of all the studies included in the meta-analysis

First author (Year)	Origin country	No. of patients	Median/mean age (year)	WHO grade	Mutant IDH No. (%)	IDH mutation type	Primary	Mutation detect method	Survival end points	Follow up period (years or months or day)	Survival analysis	Adjusted variables	Adjusted or matched critical variables from the EORTC				
													Age/age>40	Tumor size (6cm)	Histology	neurologic deficit	crossing midline
Hartmann, 2013	Germany	69	49(21–74)	IV	IDH1 22/67 (32.8%);IDH2 1/65 (1.5%)	IDH1/2	YES	direct sequencing	OS	73M	Univariate	NR	-	-	-	-	-
Sabha, 2014	Canada	108	NR	II–III	IDH 93/108 (86%)	IDH1/2	YES	direct sequencing	OS,PFS	OS: 70 (18–144)M PFS: 65(18–168)M	Multivariate	1p/19q status, PTEN status	-	-	-	-	-
Zhang, 2014	China, Xi'an	240	NR	II-IV	IDH1 103/240 (42.92%)	IDH1	YES AND NO	NR	OS,PFS	NR	Multivariate	sex,WHO grade,age, KPS, histology, Methylation in tumor,Methylation in serum	+	-	+	-	-
Zhang, 2014	China, Beijing	203	NR	II–III	IDH1 108/203 (53.20%);IDH2 2/203 (1.47%)	IDH1/2	YES AND NO	DNA pyro-sequencing	OS,PFS	NR	Multivariate	MGMT,MGMT,Ki-67, EGFR,Mutant P53,MMP-9,PTEN,and tumor locations	-	-	-	-	-
Wang, 2014	France	1305	II 38.1(16.1-77.0) III47.8(19.1-89.1) IV 58.5(18.1-89.2)	II–IV	IDH1 609/1305 (46.7%);IDH2 30/980 (3.1%)	IDH1/2	YES AND NO	direct sequencing	OS,PFS	NR	Multivariate	Age,Surgery extent, 1p19q codeletion, IK,P16 deletion	+	-	-	-	-
Stancheva, 2014	Bulgarian	106	56	IV	IDH1 13/106 (12.3%);IDH2 1/106 (0.9%)	IDH1/2	YES AND NO	direct sequencing	OS	NR	Multivariate	Age, Gender, Secondary versus primary GBM, Resection, KPS,IDH1/IDH2, TP53	+	-	-	-	-
Shibahara,2012	Japan	115	46 (10-77)	III	IDH1 70/115 (61%);IDH2 6/115 (5.2%)	IDH1/2	YES AND NO	direct sequencing	OS,PFS	43 (1-254) M	Multivariate	IDH1/2, Histological diagnosis, Ki-67 labeling index, EGFR	-	-	+	-	-
Polivka, 2014	Czech Republic	44	64.3 (35–87)	IV	IDH1 20/44(45.5%)	IDH1	YES AND NO	direct sequencing	OS,PFS	NR	NR	NR	-	-	-	-	-
Molenaar, 2014	Netherlands	98	55(27–80)	IV	IDH1 18/98 (18%) IDH2 0/98(0%)	IDH1/2	YES AND NO	direct sequencing	OS	384day (15day-7.5Y)	Multivariate	Age,Extent of resection,KFS,Radiotherapy,Secondary glioblastoma,CDKN2A alteration,EGFR alteration,IDH1 mutation,MGMT methylation,PIK3CA mutation,TP53 mutation,PTEN mutation,1p19q codeletion	+	-	-	-	-
Minniti,2014	Italy	94	43(20-71)	III	IDH1 51/94 (42.92%)	IDH1	YES AND NO	Immunohistochemistry	OS,PFS	46(12–89)M	Multivariate	IDH1,MGMT,KPS, Age,Extent of resection	+	-	-	-	-
Hatanpaa,2014	USA	50	37.5(20-66)	II–III	IDH 42 (84 %)	IDH1/2	YES AND NO	Immunohistochemistry	OS	OS:4.3Y PFS:5.1y	Multivariate	Nestin, IDH 1/2 mutation status, WHO grade, oligodendroglioma component (astrocytoma or oligoastrocytoma), MIB-1 proliferation index, age at diagnosis, sex, extent of resection (gross total vs. partial or biopsy), and type of adjuvant therapy (combined temozolomide and radiation vs. other).	+	-	+	-	-
Goze, 2014	French	131	38 (15-66)	II	IDH1 107(81.7%).	IDH1	YES AND NO	Immunohistochemistry	OS,PFS	55 (3.6–262) M	Multivariate	Sex, Age, Increased intracranial pressure, Neurological deficit, Seizures, KPS, Imaging parameters, Histopathological parameters, 1p19q Codeletion, p53 Overexpression, IDH1 expression, Therapeutic parameters,	+	+	+	+	-
Cai, 2014	China	160	18-81	II–IV	IDH 66/160(41.25%)	IDH1/2	YES AND NO	Pyrosequencing	OS	NR	Multivariate	Age, gender, KPS, Classification, Grade, ATRX,Ki-67,extent of resection, radiotherapy, chemotherapy	+	-	-	-	-
Metellus, 2010	France	47	39 (21-71)	II	IDH 40/47(85.11%)	IDH1/2	YES	DNAsequencing, Immunohistochemistry	OS,PFS	62 (7–112) M	Multivariate	Age,KPS score,FTI tumor location,Largest diameter (MRI based),Infiltration score (MRI based),Extent of surgery,1p19q deletion,IDH mutation	+	+	-	-	-
Christensen, 2010	USA	95	NR	II–IV	IDH 57/95(60%)	IDH1/2	YES AND NO	Direct sequencing	OS	NR	Multivariate	Age at diagnosis, sex, and grade-specific histology	+	-	+	-	-
Nobusawa, 2009	Switzerland	407	59.5±13.9	IV	IDH1 36/407(8.8%).	IDH1	NO	Direct sequencing	OS	NR	Multivariate	Age and gender	+	-	-	-	-
Thon,2012	Germany	127	37 (18-75)	II	IDH1 89/114 (78.1%)	IDH1	YES AND NO	Pyrosequencing	OS,PFS	OS:118 (96-130)M PFS:51 (41-63)M	Multivariate	Increased age, continuously scaled,IDH1,TP53	+	-	-	-	-
Takano, 2012	Japan	164	48.6 (18-83)	II–IV	NR	IDH1	YES AND NO	Immunohistochemistry	OS,PFS		Multivariate	Age, location, KPS, Total removal, radiation first, VEGF, IDH1,tumor size, Pathology, INA, MIB-1, p53	+	+	-	-	-
Gravendeel,2009	USA	226	50.2 (11.7-81.2)	I-IV	NR	IDH1	YES AND NO	Direct sequencing	OS	NR	Multivariate	Molecular cluster, Histologic type, Histologic grade, KPS, Age at diagnosis, Sex, Extent of surgery, Chemotherapy, EGFR amplification, LOH 1p, LOH 19q, IDH1 mutation	+	-	+	-	-
Sanson, 2009	France	404	48.6 (16-84)	II–IV	IDH1 155/404 (38.37%)	IDH1	YES AND NO	Direct sequencing	OS,PFS	NR	Multivariate	presence of 1p19q codeletion, EGFR amplification, MGMT methylation, histologic grade, age, type of surgery, and postoperative treatment.	+	-	-	-	-
Kim, 2010	Switzerland, Germany , Japan, France	268	NR	II	IDH1 239/268 (88.80%);	IDH1/2	YES	Single-strand conformational polymorphism analysis	OS	NR	Multivariate	Age, histological grade, treatment, and genetic alterations, (including EGFR amplification, MGMT methylation, and co-deletion of 1p/19q, but not TP53 mutation	+	-	-	-	-
Hartmann, 2011	Germany	(cohort A)40 (cohort B)50	NR	II	NR	IDH1	YES	Direct sequencing	OS,PFS	NR	Multivariate	Age, diagnosis, Resection, Molecular markers,therapy	+	-	-	-	-
Weller, 2009	Germany.	301	61.5 (19.2-86.6)	IV	IDH1 16/286(5.59%)	IDH1	YES AND NO	Direct sequencing	OS,PFS	NR	Multivariate	age at diagnosis, KPS, extent of resection,MGMT promoter methylation status, and adjuvant therapy	+	-	-	-	-
Mukasa,2012	Japan	250	NR	II–IV	IDH1 73/250 (29%);IDH2 2/200 (1%)	IDH1/2	YES AND NO	Direct sequencing	OS,PFS	NR	Multivariate	IDH mutation,1p/19q co-deletion,TP53 mutation,Methylated MGMT promoter	-	-	-	-	-
Li, 2011	China	47	43 (16-71)	III	IDH1 239/47 (53.19%);	IDH1	NR	Direct sequencing	OS	38.2 (14.0–49.5) M	Multivariate	Age, LOH 1p and 19q, P53 expression, Reoperation after progression, Adjuvant chemotherapy	+	-	-	-	-
Elsir, 2011	Sweden	116	40	II	IDH1 90/116(77.59%)	IDH1	NR	Immunohistochemistry	OS	14.5 (10.0–22.5)M	Multivariate	Clinical variables(Age at onset,Performance status,Tumour size,Tumour crossing midline,Contrast enhancement,Histology,Extent of resection),Molecular variables(Ki-67 expression,IDH1 mutation,PROX1-positive cells)	+	+	+	-	+
Ohka,2011	Japan	57	42(21-72)	II	IDH1 47/57(82.46%)	IDH1	YES AND NO	Pyrosequencing	OS,PFS	NR	Multivariate	Age, Sex, surgery,adjuvant therapy,1p/19q codeletion,MGMT status,LINE-1 methylation	+	-	-	-	-
Yao,2013	China	53	38(5-67)	II–IV	IDH1 47/57(82.46%)	IDH1	YES AND NO	Direct sequencing	OS,PFS	NR	Multivariate	Age, WHO grade, tumor phenotype and IDH1status	+	-	-	-	-
Yang,2013	China	206	53.6(14-78)	IV	NR	IDH1	YES AND NO	Direct sequencing	OS	12.3 (1–60)M	Multivariate	Sex, age at diagnosis, KPS score, Tumor size,WHO grade, extent of resection, MGMT promoter methylation and IDH1 mutation,CD147 expression	+	+	-	-	-
Takahashi,2013	Japan	267	NR	III-IV	NR	IDH1	YES AND NO	Direct DNA sequencing	OS	NR	Multivariate	Age, gender, resection, IDH1 mutation status, MGMT status existence of 1p19q co-deletion, Histology	+	-	+	-	-
Shin,2013	Korea	67	NR	IV	IDH1 10/67 14.9%	IDH1	YES AND NO	Immunohistochemical	OS,PFS	NR	Multivariate	Age,Sex,Extent of surgery,Tx of TMZ,Tx of CCRT,CD133,CD34,VEGF	+	-	-	-	-
Yang, 2012	China	315	45.9±12	I–IV	IDH1 112/315(35.56%)	IDH1	YES AND NO	Direct sequencing	PFS	22.50 (14 - 39) M	Multivariate	Sex, age at diagnosis, KPS score, WHO grade, extent of resection, and IDH1 mutation, brian Deep structure involved	+	-	--	-	-
Wang, 2012	China	131	NR	IV	IDH1 29/131(22.1%)	IDH1	YES AND NO	Pyrosequencing	OS,PFS	NR	Univariate	Sex, age at diagnosis, KPS score, resection, RT plus CT (vs. RT alone), and IDH1 mutation, MGMT genemethylation	+	-	-	-	-
van den Bent,2010	EORTC	159	NR	II–IV	IDH1 73/159(46%)	IDH1	YES AND NO	Bidirectional cycle sequencing of PCR-amplified fragments	OS	NR	Multivariate	IDH1 mutations, of necrosis, and of 1p/19q loss	-	-	-	-	-
Tayrac,2011	French	189	57(13–80)	III–IV	IDH1 30/189(15%)	IDH1	YES AND NO	Direct sequencing	OS	minimum 2 Y	Multivariate	Age,treatment, grade, RTOG RPA classes, MGMT methylation status, and IDH1 mutational status	+	-	-	-	-
Taal,2011	Netherlands	42	38(25–59)	I–IV	IDH1 36/42(86%)	IDH1	YES AND NO	Bidirectional cycle sequencing of PCR-amplified fragments	OS,PFS	14(4-104)M	Multivariate	Trisomy 7,TP53,MGMT status,19q loss,IDH1	-	-	-	-	-
Philippe, 2011	France	89	39(21-71)	II	IDH1 74/89(83.15%)	IDH1	YES	NR	OS	NR	Multivariate	age, FTI tumor locaton, Infitration score,Extent of surgery,IDH mutation	+	-	-	-	-
Lotsch,2013	Austria	100	60(15-85)	IV	IDH1 6/100(6%)	IDH1	YES AND NO	Direct sequencing	OS	NR	Univariate	Age, KPS, treatment, and IDH1 mutation status,hTERT mRNA expression,Telomere length,MGMT promoter methylation	-	-	-	-	-
Leu,2013	France	210	41.9	II	IDH1 91/130(70.3%)	IDH1	YES AND NO	Pyrosequencing	OS	NR	Multivariate	Age, gender, epilepsy, focal neurological deficit, 1p19qloh, tp53pos, MGMTmet	+	-	-	+	-
Okita, 2012	Japan	58	39 (21-75)	II	IDH 34/58(58.62%)	IDH1/2	YES AND NO	Bidirectional cycle sequencing of PCR-amplified fragments	OS,PFS	NR	Multivariate	Histology, IDH mutation, Age (years), Extent of removal, Initial KPS	+	-	+	-	-
Ahmadi, 2012	Germany	100	37.4(19.8-72.1)	II	IDH1 79/100(79%)	IDH1	YES AND NO	direct sequencing	OS,PFS	91.1(84.6–113.2)M	Multivariate	Age, KPS, extent of resection, radiotherapy, and chemotherapy	+	-	-	-	-
Dahlrot, 2013	Denmark	52	48(18–82)	II	IDH1 30/52(57.69%)	IDH1	YES AND NO	Immunohistochemistry	OS,PFS	36(0.4–79)M	Multivariate	Age,Gender,Enhancement,Midline shift,Crossing midline,Localization,Neurologic deficit,Headache,Epilepsy,Mental disturbance,Surgery,Post-surgical treatment,Performance status,Histology,1p/19q deletion,IDH mutation	+	-	+	+	+
Chou, 2012	USA	124	48.5(19-79)	IV	IDH1 23/124(18.55%)	IDH1/2	YES	Bidirectional cycle sequencing of PCR-amplified fragments	OS	NR	K-M	NR	-	-	-	-	-
Boots-Sprenger	Netherlands, USA	442	NR	II–IV	IDH1 209/442(47.29%)	IDH1	YES AND NO	Direct sequencing	OS	NR	K-M	NR	-	-	-	-	-
Juratli, 2012	Germany	99	44.3(23.3-76.7)	III-IV	IDH1 75/99(75.76%)	IDH1/2	NO	Direct sequencing	OS,PFS	over 9 Y	Multivariate	Age, Diagnosis of malignancy, ,IDH mutation status. ,MGMT promoter status, CT and RT at malignancy	+	-	+	-	-
Myung,2012	Korea	134	41.3 (3-71)	II-IV	IDH1 72/134(53.7%)	IDH1	YES AND NO	Direct sequencing	OS,PFS	9.9-130.5	K-M	NR	-	-	-	-	-
Combs, 2011	Germany	140	56(20-76)	IV	IDH1 4/140(3%)	IDH1	YES	Immunohistochemistry	OS	NR	K-M	NR	-	-	-	-	-
Qi,2011	China	203	36.4(2-78)	II–IV	IDH 80/203(39.41%)	IDH1/2	YES AND NO	PCR	OS,PFS	22.6(2.9-79.9)M	K-M	NR	-	-	-	-	-
Schittenhelm, 2011	Germany	41	51(18–81)	III	IDH1 21/41(51.22%)	IDH1	YES	Immunohistochemistry	OS	2.3 Y	K-M, multivariate	age	+	-	-	-	-
Gorovets,2012	USA	90	NR	III	IDH 62/90(68.89%)	IDH1/2	YES	Immunohistochemistry	OS	NR	K-M	NR	-	-	-	-	-
Lewandowska,2014	Polish	139	36 (18-76)	I-IV	IDH1 87/139(62.59%)	IDH1	YES AND NO	Immunohistochemistry, Sanger Sequencing, and COLD PCR HRM	OS	NR	K-M	NR	-	-	-	-	-
Kizilbash.2014	USA	141	NR	III	IDH 60/141(42.55%)	IDH1/2	YES AND NO	Pyrosequencing	OS	36.4M	Multivariate	Concurrent TMZ, age, sex, Subtotal or gross total resection, IDH mutation, age, Frontal, parietal or occipital, tumor size and year of diagnosis	+	+	-	-	+
Collins, 2014	UK	281	NR	III-IV	IDH 82/281(29%)	IDH1/2	NO	Pyrosequencing	OS	NR	Multivariate	Age, Gender, Tumor grade, WHO Performance status	+	-	-	-	-
Baldock, 2014	USA	172	NR	II-IV	IDH1 16/172(9.3%)	IDH1	YES AND NO	Immunohistochemistry	OS	NR	K-M	NR	-	-	-	-	-
Yan, 2009	USA	171	NR	III-IV	IDH 52/177(29.38%)	IDH1/2	YES AND NO	Direct sequencing	OS	NR	K-M	NR	-	-	-	-	-

Supplementary Table S2: General statistics of OS and PFS statistics of included studies
